# Changes of blood-brain-barrier function and transfer of amyloid beta in rats with collagen-induced arthritis

**DOI:** 10.1186/s12974-021-02086-2

**Published:** 2021-01-30

**Authors:** Po-Hsuan Lai, Ting-Hsuan Wang, Nai-You Zhang, Kuo-Chen Wu, Chung-Chen Jane Yao, Chun-Jung Lin

**Affiliations:** 1 School of Pharmacy, College of Medicine, National Taiwan University, 33 Linsen South Road, Taipei, Taiwan; 2 Graduate Institute of Clinical Dentistry, Dental School, College of Medicine, National Taiwan University, Taipei, Taiwan

**Keywords:** Collagen-induced arthritis, Blood-brain barrier, Amyloid beta, Receptor of advanced glycation end product, P-glycoprotein

## Abstract

**Background:**

Rheumatoid arthritis (RA) is characterized by synovial inflammation, cartilage damage, and systemic inflammation. RA is also associated with the occurrence of neuroinflammation and neurodegenerative diseases. In this study, the impacts of RA on the function of the blood-brain barrier (BBB) and the disposition of amyloid beta (Aβ), including BBB transport and peripheral clearance of Aβ, were investigated in rats with collagen-induced arthritis (CIA), an animal model with similarity to clinical and pathological features of human RA.

**Methods:**

CIA was induced in female Lewis rats. In addition to neuroinflammation, the integrity and function of the BBB were examined. The expression of Aβ-transporting proteins at brain blood vessels was measured. Blood-to-brain influx and plasma clearance of Aβ were determined.

**Results:**

Both microgliosis and astrogliosis were significantly increased in the brain of CIA rats, compared with controls. In terms of BBB function, the BBB permeability of sodium fluorescein, a marker compound for BBB integrity, was significantly increased in CIA rats. Moreover, increased expression of matrix metalloproteinase-3 (MMP-3) and MMP-9 and decreased expression of tight junction proteins, zonula occludens-1 (ZO-1) and occludin, were observed in brain microvessels of CIA rats. In related to BBB transport of Aβ, protein expression of the receptor of advanced glycation end product (RAGE) and P-glycoprotein (P-gp) was significantly increased in brain microvessels of CIA rats. Notably, much higher expression of RAGE was identified at the arterioles of the hippocampus of CIA rats. Following an intravenous injection of human Aβ, significant higher brain influx of Aβ was observed in the hippocampus of CIA rats.

**Conclusions:**

Neuroinflammation and the changes of BBB function were observed in CIA rats. The increased RAGE expression at cerebral blood vessels and enhanced blood-to-brain influx of Aβ indicate the imbalanced BBB clearance of Aβ in RA.

**Supplementary Information:**

The online version contains supplementary material available at 10.1186/s12974-021-02086-2.

## Background

Rheumatoid arthritis (RA) is a chronic and systemic inflammatory disorder that is characterized by synovial inflammation, cartilage damage, and systemic inflammation. Proinflammatory cytokines, such as interleukin-1β (IL-1β), tumor necrosis factor-alpha (TNF-α), and interleukin-6 (IL-6), are the important inflammation-related mediators regulating the disease state of RA [7]. These proinflammatory cytokines can systemically regulate the expression and/or activities of various proteins, including drug-metabolizing enzymes and membrane transporters [21, 49]. The systemic inflammation occurred in RA may also play an important role in the pathogenesis of neurodegenerative diseases [30] and cognitive impairment [10]. While the systematic review and population-based study have reported a higher risk of cognitive decline, including cognitive impairment and dementia/Alzheimer’s disease (AD), in patients with RA [5, 23, 45], others have revealed inverse relationship [31]. Nevertheless, it is noted that the association between RA and AD can be confounded by factors such as age, disease diagnosis and status, and medication [31].

Despite that the association between RA and AD remains to be verified, the disruption of the blood-brain barrier (BBB) has been reported in collagen-induced arthritic DBA/1 mice at the advanced and later stages (21-100 days after immunization) of the arthritis [28]. The BBB is constituted by a specialized microvascular endothelium that interacts with pericytes, astrocytes, and neurons and is an important interface between brain and blood compartments. While the paracellular permeability of molecules at the BBB is generally restricted, there are several pathways that can mediate the influx and efflux of substances across the BBB. In related to AD, β-amyloid (amyloid beta or Aβ) can move across the BBB [11]. As Aβ is produced in both the brain and the periphery, the transport of Aβ across the BBB is bidirectional (i.e., influx or efflux) and can be mediated by receptor-mediated pathways and transporter-mediated pathways. For receptor-mediated pathways, RAGE (receptor for advanced glycation end products) appears to mediate the influx of Aβ from the blood to the brain, whereas LRP-1 (low-density lipoprotein receptor-related protein 1) mediates the efflux of Aβ from the brain to the blood [43, 52]; for transporter-mediated pathways, several ATP-binding cassette transporters (ABC transporters) are involved in Aβ transport. Among them, both Aβ40 and Aβ42 have been demonstrated to be the substrates of P-glycoprotein (P-gp; ABCB1) [18].

While BBB is important for the clearance of Aβ in the brain, it is unclear whether RA can change the transport of Aβ across the BBB. Nevertheless, in-vitro assays have shown that the expression of LRP-1 can be downregulated in cultured human microvascular endothelial cells with the treatments of cytokines [17] and LPS [13]. Also, the expression of P-gp at the BBB can be regulated by NF-κB and TNF-α dependent pathways [3, 15]. These findings suggest that Aβ transport at the BBB may be altered in RA by inflammation-related pathways. In this regard, the present study was aimed to investigate the impacts of RA on the disposition of Aβ, including BBB transport and peripheral clearance of Aβ, in rats with collagen-induced arthritis (CIA), an animal model with similarity to clinical and pathological features of human RA.

## Methods

### Induction of CIA rats

Female Lewis rats (LEW/SsNNarl; 5 weeks old) were purchased from BioLASCO Taiwan (Taipei, Taiwan) and kept in National Taiwan University College of Medicine Laboratory Animal Center. All animals were maintained under standard conditions with a 12-h dark/light cycle and acclimatized for 1 week before the experiment. Food and water were available ad libitum. CIA rats were induced according to the methods described previously [21]. In brief, an aliquot of 250 μL of the emulsion, containing equal parts of Freund’s complete adjuvant and 2 mg/mL bovine type II collagen, was subcutaneously injected to the base of the tail of the rats. Seven days after the first immunization, a booster dose of 250 μL of the same emulsion was injected again. On the other hand, control rats were injected with 250 μL normal saline at the base of the tail. CIA rats and controls were weighed and the volumes of paws were measured. On day 17 following the first immunization, plasma and brain samples were collected and the levels of proinflammatory cytokines were measured. Protein levels of cytokines were determined by enzyme-linked immunosorbent assay (ELISA; IL-1β and IL-6: 900-M91, 900-M-86, Peprotech, NJ, USA; TNF-α: 438204, BioLegend, SD, USA) according to manufacturer’s instructions; mRNA levels of cytokines were determined by RT-qPCR.

### RT-qPCR analysis

Total RNA was isolated from the cortex, hippocampus, and striatum with TRI-200 reagent (Bioman Science Co., Ltd., Taiwan). cDNA was prepared from 1 μg RNA of each sample with 0.5 μg Oligo dT_12-18_ (Arrowtec, Taiwan) and GoScript^TM^ reverse transcription system (Promega, Madison, WI, USA) according to manufacturer’s instructions. An aliquot of 1 μL of cDNA was mixed with 7 μL of sterile deionized water, 10 μL of Power SYBR Green PCR master mix (Applied Biosystem, UK), and 1 μL of forward/reverse primers (10 μM each; Supplementary Table 1). The program for PCR started from the denaturation at 95 °C for 10 min, followed by 40 amplification cycles of 95 °C for 15 s, and then 56 °C for 15 s. The process and signal collection were performed on CFX Connect Real-Time PCR Detection System (Bio rad, Hercules, CA, USA). The specificity of PCR products was confirmed by the dissociation curve analysis. The relative quantity of target gene normalized with *Gapdh* was calculated by comparative Ct (ΔCt) method. ΔΔCt value was obtained by subtracting the ΔCt value of each CIA rat from that of the control rat and the relative amount was determined by the formula: 2^−ΔΔCt^.

### Immunofluorescence

On day 17 following the first immunization, rats were anesthetized by intraperitoneal injection of ketamine (50 mg/kg) and xylazine (10 mg/kg) and were intracardially perfused with ice-cold normal saline and then with 4% paraformaldehyde. The brains were post-fixed with 4% paraformaldehyde and then immersed in 30% sucrose solution. Before being sliced, the brains were embedded in optimal cutting temperature (OCT) compound (Sakura Finetek, Torrance, CA, USA) at −20 °C. The non-specific binding was blocked with 2.5% skim milk containing 0.1% Triton X-100. For the identification of zonula occludens-1 (ZO-1), P-gp, and RAGE, the antigen retrieval was required to expose antigenic sites and the brain slices were heated with retrieval buffer (pH 9; Dako, Carpinteria, CA, USA) at 80 °C for 40 min. Brain sections were immune-stained with the following primary antibodies: mouse anti-glial fibrillary acidic protein (Gfap) (1/1000 dilution; Cell signaling technology, Ma, USA), rabbit anti-ionized calcium-binding adapter molecule-1 (Iba-1) (1/1200 dilution; Wako, Tokyo, Japan), rabbit anti-ZO-1 (1/200 dilution; Life Technologies, Carlsbad, CA, USA), mouse anti-P-gp (1/200; BioLegend, SD, USA), rabbit anti-matrix metalloproteinase-3 (MMP-3) (1/500, Abcam, Cambridge, MA, USA), rabbit anti-MMP-9 (1/500, Abcam), or mouse anti-RAGE (1/200; Santa Cruz Biotechnology, CA, USA), all diluted in 0.5% skim milk at 4 °C. The immunolabeling was visualized using secondary antibodies labeled with rhodamine or AlexaFluor 488 (Jackson ImmunoResearch Laboratories, PA, USA) diluted in phosphate-buffered saline (PBS). For double immunofluorescence, the sections were incubated with a mixture of mouse anti-RAGE (1:200; Santa Cruz Biotechnology) and goat anti-alpha smooth muscle actin (α-SMA) (1:250; Abcam), or a mixture of mouse anti-RAGE (1:200; Santa Cruz Biotechnology) and goat anti-CD31 (1:250; R&D Systems, Minneapolis, MN, USA), followed by the incubation with goat anti-mouse rhodamine-conjugated and chicken anti-goat fluorescein isothiocyanate-conjugated secondary antibodies.

The images were acquired using Zeiss Axio Imager M1 fluorescence microscope (Zeiss, Oberkochen, Germany). The mean intensity was obtained from the integrated density divided by area, and the value of fluorescence was quantified by the ImageJ v1.51 software (National Institutes of Health, Bethesda, Maryland, USA). The activation of Iba-1 positive microglia score was calculated based on the morphology [32]. The scores ranged from zero to three, representing ramified, intermediate, reactive, and ameboid, respectively. Score 0, round and small soma with thin processes; score 1, a little bigger and more elongated soma with thicker processes; score 2, larger soma with less and thicker processes; score 3, more severe soma enlarged. The investigators were blinded to the experimental conditions (CIA or control group) during the assessment of Iba-1 scores. The number of each score was quantified and shown as percentage.

### Measurement of BBB permeability

Rats were anesthetized and catheterized with PE-50 polyethylene tube at the right femoral vein. Sodium fluorescein (2%) (Sigma-Aldrich, St. Louis, MO, USA) in normal saline was injected through the catheter at a dose of 5 mL/kg [16]. Thirty minutes after the injection, rats were perfused transcardially with ice-cold normal saline. Brain tissues were collected and homogenized in PBS with equal volume of 60% trichloroacetic acid. The homogenate was centrifuged at 18,000×*g* at 4 °C for 10 min. The fluorescence of the supernatant was measured using 440 nm excitation and 525 nm emission filters. Extraction ratio was calculated by the following equation: ([tissue fluorescence]/[g brain])/([plasma fluorescence]/[mL blood]) × 100% [34].

### Measurement of MMPs activity

The enzymatic activity of MMPs was determined using the SensoLyte®520 Generic MMP assay kit according to manufacturer’s instructions (#71158; AnaSpec, San Jose, CA.) In brief, brain samples were homogenized in assay buffer containing 0.1% Triton X-100 and were then centrifuged at 10,000×*g* for 15 min at 4 °C. The supernatants were incubated with equal volume of 2 mM 4-aminophenylmercuric acetate at 37 °C for 3 h and 35 μg of total protein were mixed with MMP substrate solution. The fluorescence signal was measured at excitation/emission = 490/520 nm using a SpetraMax Paradigm microplate reader (Molecular Devices, LLC., Sunnyvale, CA, USA).

### Isolation of brain microvessels

Brain microvessels were isolated according to the procedure described previously [50]. For each experiment, the brains of two rats were homogenized in ice-cold Hank’s buffered salt solution (HBSS; 14065-056; Gibco, USA) (4 mL per gram of the tissue) using Glas-Col homogenizer by 20 up-and-down strokes at 400 rpm. The homogenate was centrifuged at 1000×*g* for 10 min. The pellet was resuspended in 15 mL 20% dextran (70 kDa, TCI, Tokyo, Japan) and then centrifuged at 4500×*g* for 15 min. The resulting pellet was dissolved in 4 mL HBSS containing 1% bovine serum albumin (BSA; Sigma-Aldrich) and the suspension was passed through a 100-μm mesh nylon filter (BD Falcon, Durham, NC, USA) and then a 20-μm mesh nylon filter (Millipore, Temecula, CA, USA). Brain microvessels retained on the filter were collected with 4 mL HBSS containing 2% proteinase inhibitors (Roche Diagnostics, Indianapolis, IN, USA), and centrifuged at 4500×*g* for 15 min. For immunoblot analysis, the pellet of microvessels was dissolved in capillary lysis buffer (150 mM NaCl, 50 mM Tris-HCl, 0.5% Triton X-100, 0.5% sodium deoxycholate, and 2% protease inhibitors) on ice. The lysate was centrifuged at 10,000×*g* for 10 min, and the supernatant was collected, aliquoted, and stored at −70 °C. Protein expression of RAGE, LRP-1, P-gp, ZO-1, and occludin was examined by Western blot analysis.

### Determination of soluble LRP-1 and hepatic LRP-1 and P-gp

Plasma levels of soluble LRP-1 (sLRP-1) were measured with ELISA (LS-F22644, LifeSpan BioScience, Seattle, WA, USA) according to manufacturer’s instructions. For hepatic LRP-1 and P-gp, liver tissue was homogenized in radioimmunoprecipitation assay buffer (RIPA; 150 mM NaCl, 50 mM Tris base, 1% NP-40, 0.5% sodium deoxycholate, and 0.1% sodium dodecyl sulfate (SDS); pH 8), incubated on ice for 30 min, and then centrifuged at 14,000×*g* for 15 min. The supernatant was collected and stored at −70 °C until analysis. To isolate membrane fraction, liver tissue was homogenized in 0.01 M Tris–HCl buffer (pH 7.4), containing a protease inhibitor cocktail. After 20 strokes in a glass-Teflon homogenizer at 400 rpm on ice, the homogenate was centrifuged at 4000×*g* for 15 min at 4 °C, and the supernatant was ultra-centrifuged (sw55ti, 342194, Beckman Coulter, Brea, CA, USA) at 100,000×*g* for 60 min at 4 °C. The pellet was resuspended in 1 mL of 0.01 M Tris–HCl buffer (pH 7.4), containing protease inhibitors, and stored at −70 °C until use. Protein expression of LRP-1 and P-gp was measured by Western blot analysis.

### Western immunoblotting

Protein concentrations were analyzed by Bio-Rad DC Protein Assay kit (Bio-Rad, Hercules, CA, USA). Protein samples (10 μg each) were diluted with loading buffer (200 mM Tris-HCl, 1.43% 2-mercaptoethanol, 0.4% bromophenol blue, and 40% glycerol) and heated at 98 °C for 10 min (for Gapdh, LRP-1, RAGE, and occludin) or for 1 min (for P-gp and ZO-1). The samples were then separated on 10% SDS-polyacrylamide gel in running buffer (0.3% Tris base, 1.88% glycine, and 0.1% SDS) and transferred onto a nitrocellulose membrane in transfer buffer (0.3% Tris base, 1.88% glycine, and 20% methanol; pH 8.3). Non-specific binding to the membrane was blocked with 5% skim milk in TNT buffer (10 mM Tris-HCl, 150 mM NaCl, and 0.2% Tween 20; pH 7.4) at room temperature. The membrane was incubated overnight at 4 °C with antibodies for LRP-1 (1:30000; Abcam, Cambridge, MA, USA), P-gp (1:150; BioLegend, SD, USA), RAGE (1:100; Santa Cruz Biotechnology, CA, USA), ZO-1 (1:300; Life Technologies, Carlsbad, CA, USA), occludin (1:300; Life Technologies, Carlsbad, CA, USA), or Gapdh (1:160000; Biodesign International, Saco, Maine, USA), all diluted in 5% skim milk in TNT buffer. The membrane was washed by TNT buffer and incubated with HRP-conjugated anti-mouse IgG antibodies (1:8000; Jackson Immuno Research Laboratories, PA, USA) or anti-rabbit IgG antibodies (1:1000; Cedarlane, Dntario, CA, USA) in TNT buffer at room temperature. Bound antibodies were detected using Chemiluminescence Reagent Plus (PerkinElmer Life Sciences, MA, USA) and Bio-Rad ChemiDoc™ XRS+ Systems and Image Lab™ Software to obtain images under appropriate exposure time.

### Plasma kinetics and brain influx of Aβ42

Before given to animals, human Aβ42 (Anaspec, Fremont, CA, USA) was disaggregated by 1,1,1,3,3,3-Hexafluoro-2-propanol (HFIP, Sigma-Aldrich) according to literature report [35]. The HFIP-treated Aβ42 (0.125 mg/mL in normal saline) was administrated through femoral vein at a dose of 0.67 mg/kg. An aliquot of 100 μL of blood samples were drawn at 3, 6, 9, 12, and 15 min after the dosing. Each blood sample was mixed with 1 μl of 15% EDTA and then centrifuged at 1300×*g* for 10 min at 4 °C. The supernatant was collected and stored at −70 °C. The plasma levels of human Aβ42 were quantified by specific human Aβ42 ELISA kits (KHB3441, Life Technologies, Carlsbad, CA, USA) according to manufacturer’s instructions. The plasma concentration–time data of Aβ were analyzed by the non-compartmental analysis. The values of the total area under the plasma concentration vs. time curve (AUC) were calculated by a linear trapezoidal rule and by extrapolating time to infinity by dividing the last measurable concentration by the value of the elimination constant. The elimination constant was estimated by the terminal plasma concentrations. The plasma clearance was estimated by the dose divided by the AUC.

For brain influx of Aβ42, 15 min after the intravenous administration, rats were perfused with ice-cold normal saline. Brain tissues (whole brain, cortex, and hippocampus) were harvested and homogenized by polytron homogenizer (HSIANGTAI, Taiwan) according to the method described by Andrew et al. [2]. The homogenate was ultracentrifuged at 100,000×*g* for an hour (sw55ti, 342194, Beckman Coulter, Brea, CA, USA) and the supernatant was preceded for solid-phase extraction according to the method described by Lanz and Schachter [20] with modifications. Before loading the supernatant into the Oasis HLB 60 mg solid-phase extraction cartridge (186000381, Waters, Milford, MA, USA), the cartridge was activated by 1 mL of methanol and equilibrated with distilled water. The supernatant was loaded and the cartridge was washed with 1 mL of 10% methanol and then with 1 mL of 30% methanol. The cartridge was eluted with 1 mL of 90% methanol containing 2% ammonium hydroxide and the eluent was vacuum-dried by SPD1010 Integrated SpeedVac™ Systems (Thermo Fisher Scientific, MA, USA). The pellet was reconstituted with a standard diluent buffer provided by the ELISA kits. The levels of human Aβ42 in the brain were quantified by specific human Aβ42 ELISA kits (KHB3544, Life Technologies, Carlsbad, CA, USA) according to manufacturer’s instructions.

### Statistical analysis

Statistical analyses were performed using GraphPad Prism Data Analysis software 6 (GraphPad Software, Inc., CA, USA). Statistical differences were evaluated by two-sample Student’s *t* test, with a level of significance of 0.05.

## Results

### Systemic inflammation and neuroinflammation in CIA rats

**Fig. 1:**
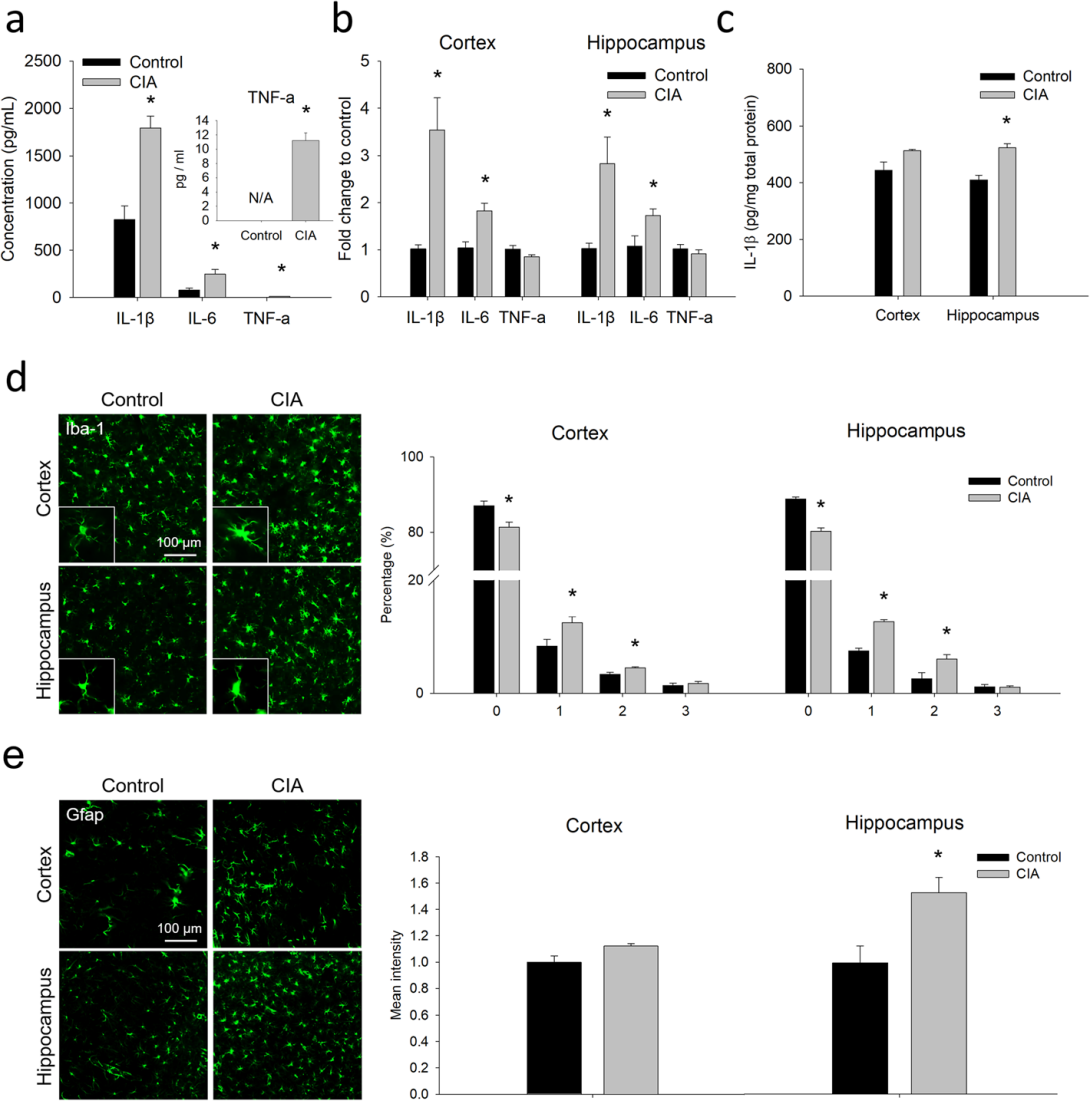
Systemic inflammation and neuroinflammation in CIA rats. **a** Plasma concentrations of IL-1β, IL-6, and TNF-α quantified by ELISA. **b** mRNA levels of IL-1β, IL-6, and TNF-α in the cortex and the hippocampus of CIA rats and controls. **c** Protein level of IL-1β in the cortex and the hippocampus quantified by ELISA. **d** Representative images of immunofluorescence of Iba-1 positive microglia in the cortex and the hippocampus of CIA rats and controls. The percentage of four morphological scores of microglia in the cortex and the hippocampus was quantified. **e** Representative images and the quantitative results of Gfap immunostaining in the cortex and the hippocampus of CIA rats and controls. The data are given as the mean ± SEM of 4-6 animals. *P < 0.05 compared with controls

The symptoms of CIA rats were characterized by the enlargement of paw volume and the change of body weight (Supplementary Figure 1). Also, plasma levels of IL-1β, IL-6, and TNF-α were significantly higher in CIA rats than in controls (Fig. 1a). In addition to the enhanced systemic cytokine levels, the expression of brain cytokine levels, microglia activation, and astrogliosis were examined. The RT-qPCR results show that the mRNA levels of IL-1β and IL-6, but not of TNF-α, were significantly increased in the cortex and the hippocampus of CIA rats (Fig. 1b). The ELISA also showed a significant higher level of IL-1β in the hippocampus of CIA rats (Fig. 1c). Significant microglia activation was observed in both the cortex and the hippocampus of CIA rats, exhibiting lower percentage of score 0 and higher percentage of score 1 and 2 in microglia morphology (Fig. 1d). On the other hand, significant astrogliosis was observed in the hippocampus, but not in the cortex, of CIA rats (Fig. 1e). These findings demonstrate the characteristics of both systemic inflammation and neuroinflammation in CIA rats.

### Reduced integrity and enhanced permeability in brain microvessels of CIA rats

**Fig. 2:**
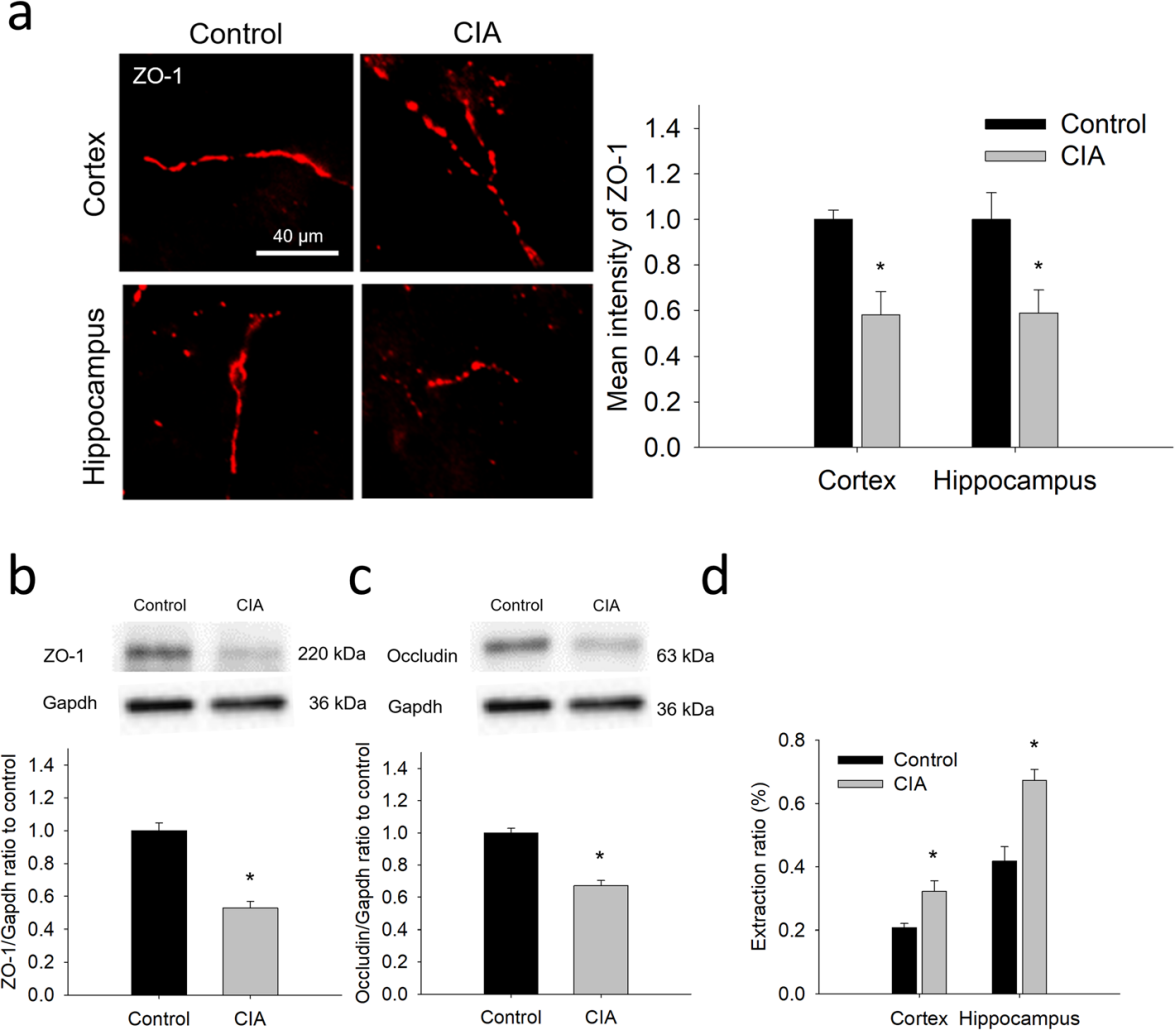
Changes in the integrity and permeability of brain microvessels of CIA rats. **a** Representative images of ZO-1 immunostaining in the cortex and the hippocampus of CIA rats and controls. The quantitative results of ZO-1 immunostaining are shown on the right-hand side of the images. **b** and **c** Immunoblotting and the quantitative densitometric analyses of ZO-1 (**b**) and occludin (**c**) in the lysate of the isolated brain microvessels. **d** BBB permeability measured by the extraction ratio of sodium fluorescein. The data are given as the mean ± SEM of 3-4 experiments. *P < 0.05 compared with controls

Given that BBB dysfunction can be associated with neuroinflammation, BBB integrity was examined in terms of the expression of tight junction proteins, ZO-1 and occludin, and the permeability of sodium fluorescein. The immunofluorescence images showed that the expression of ZO-1 was decreased in brain microvessels in the cortex and the hippocampus of CIA rats (Fig. 2a). Likewise, the immunoblots showed a significant lower expression of ZO-1 and occludin in brain microvessels isolated from CIA rats (Fig. 2b and c). In addition to the expression of tight junction proteins, 30 min after an intravenous injection of sodium fluorescein, the extraction ratios of sodium fluorescein were significantly higher in the cortex and the hippocampus of CIA rats (Fig. 2d).

**Fig. 3:**
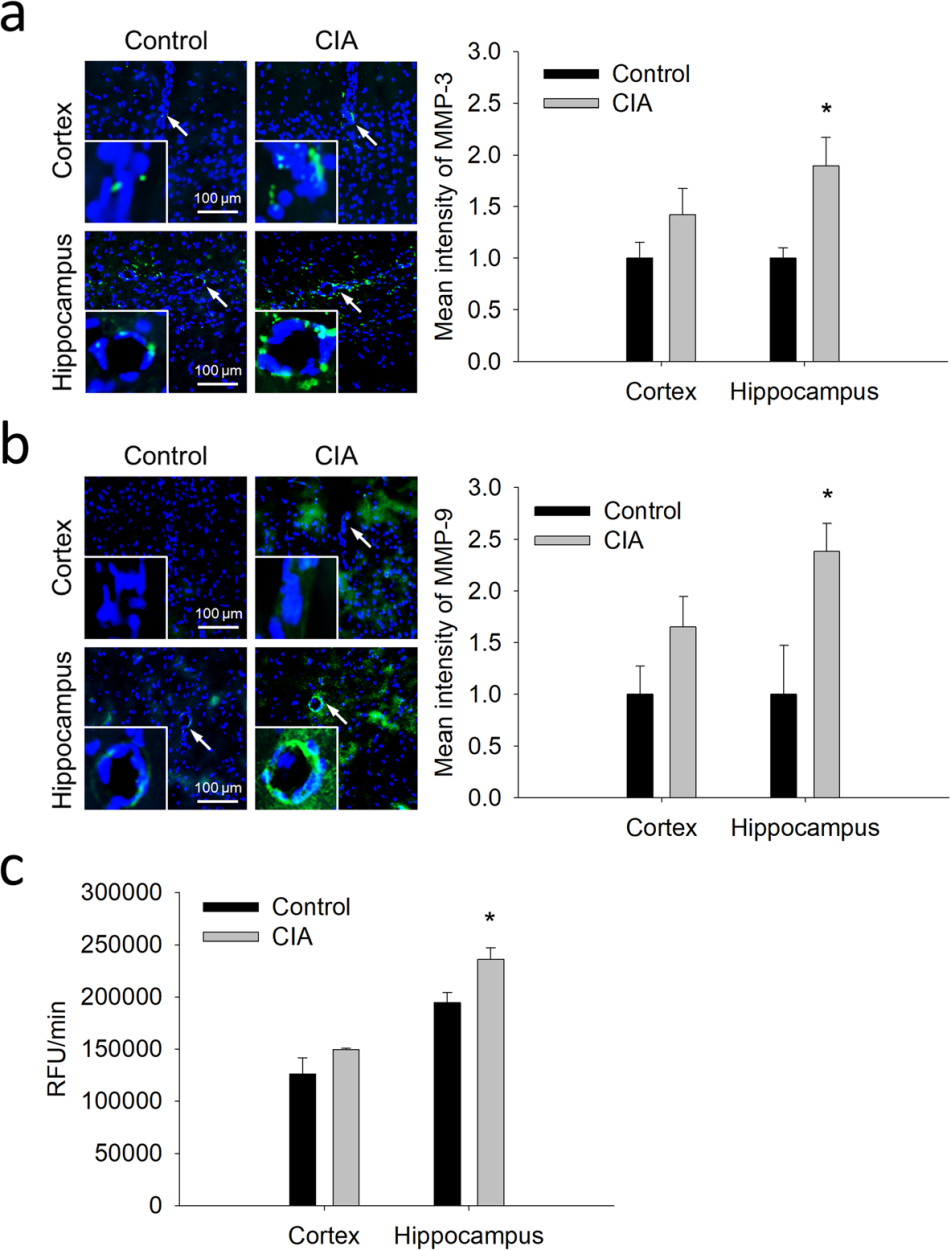
Increased expression of MMP-3 and MMP-9 at brain microvessels of CIA rats and the general MMPs activity. **a** and **b** Representative images of the immunostaining of MMP-3 (**a**) and MMP-9 (**b**) in the cortex and the hippocampus of CIA rats and controls. The quantitative results of the immunostaining are shown on the right-hand side of the images. **c** The general activity of MMPs in the cortex and the hippocampus, represented by the relative fluorescence unit (RFU) per minute. The data are given as the mean ± SEM of 3-4 animals. *P < 0.05 compared with controls

As one of the most significant contributors to BBB breakdown is the activation of proteinases such as MMPs [48], in which both MMP-3 and MMP-9 are triggered by cytokines (i.e., TNF-α and IL-1β) [51], the expression of MMP-3 and MMP-9 was examined. The results showed the expression of both MMP-3 and MMP-9 was significantly increased in the perivascular area of the hippocampus (Fig. 3a and b), but not of the cortex. Nevertheless, there was a trend of increased expression of these MMPs in the cortex. In addition to protein expression, the general activity of MMPs was examined and the results showed higher MMPs activity in the hippocampus of CIA rats (Fig. 3c). These results suggest the link between these MMPs and the BBB breakdown in CIA rats, especially in the hippocampus.

### Changes in the expression of Aβ-transporting proteins at brain microvessels in CIA rats

**Fig. 4:**
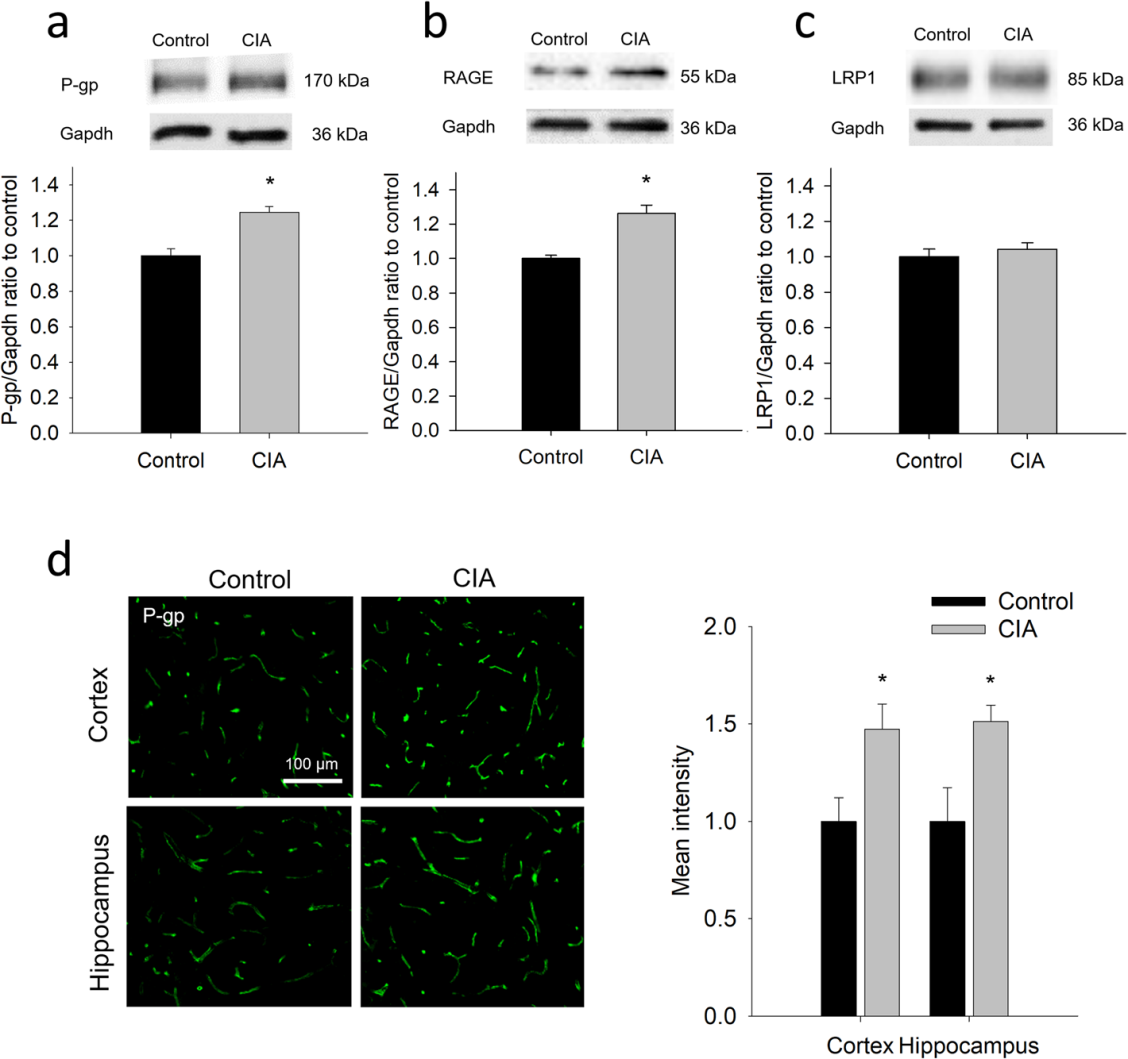
Changes in the expression of Aβ-transporting proteins at brain microvessels of CIA rats. **a**-**c** Immunoblotting and the quantitative densitometric analyses of P-gp (**a**), RAGE (**b**), and LRP-1 (**c**) in the lysate of brain microvessels isolated from CIA rats and controls. **d** Representative images and the quantitative intensity of P-gp immunostaining in the cortex and hippocampus of CIA rats and controls. The data are given as the mean ± SEM of 3-4 experiments. *P < 0.05 compared with controls

In addition to BBB integrity, the expression of proteins mediating the BBB transfer of Aβ was examined. The immunoblotting showed that protein levels of P-gp and RAGE were significantly increased in brain microvessels isolated from CIA rats (Fig. 4a and b), whereas the expression of LRP-1 (Fig. 4c) remained similar between these two groups. Similar to the findings of Western blotting, immunofluorescence analysis also showed a significant increase of P-gp expression in brain microvessels in both the cortex and the hippocampus (Fig. 4d).

**Fig. 5:**
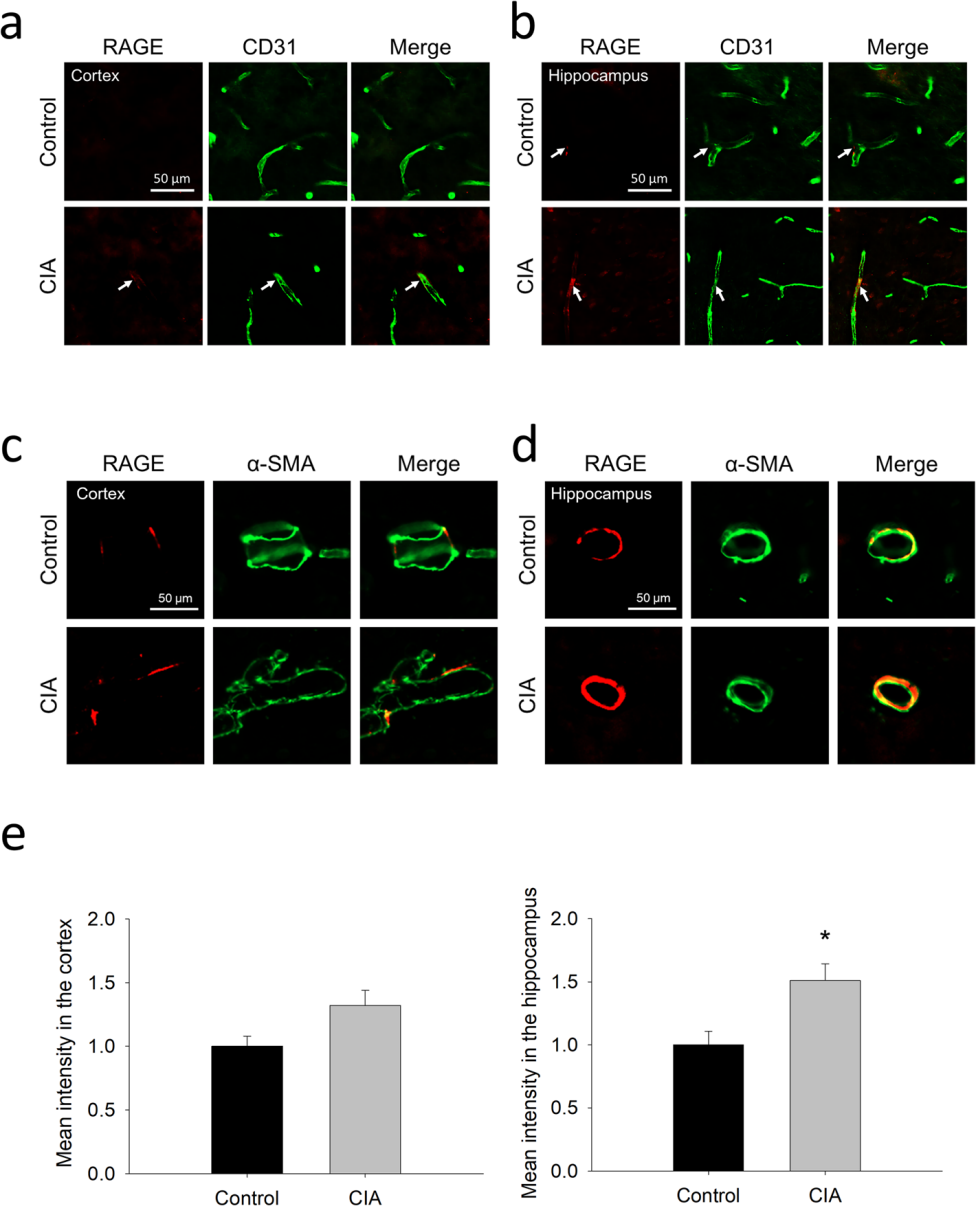
Increased expression of RAGE at cerebral blood vessels of CIA rats. **a** and **b** Representative images of the immunostaining of RAGE and CD31 (a marker of capillaries) in the cortex (**a**) and hippocampus (**b**) of CIA rats and controls. **c** and **d** Representative images of the immunostaining of RAGE and α-SMA (a marker of arterioles) in the cortex (**c**) and hippocampus (**d**) of CIA rats and controls. **e** The quantitative results of RAGE immunostaining in the blood vessels of the cortex and hippocampus. The data are given as the mean ± SEM of 3-4 animals. *P < 0.05 compared with controls

Although the data of the isolated microvessels showed an increased expression of RAGE (Fig. 4b), the expression of RAGE was low in CD31 positive microvessels of the cortex and the hippocampus (Fig. 5a and b). Compared to that in CD31 positive microvessels, RAGE expression was much higher in the arterioles that express α-SMA, especially in the hippocampus, and was significantly higher in CIA rats than in the controls (Fig. 5c and d).

### Increased blood-to-brain influx of Aβ in CIA rats

**Fig. 6:**
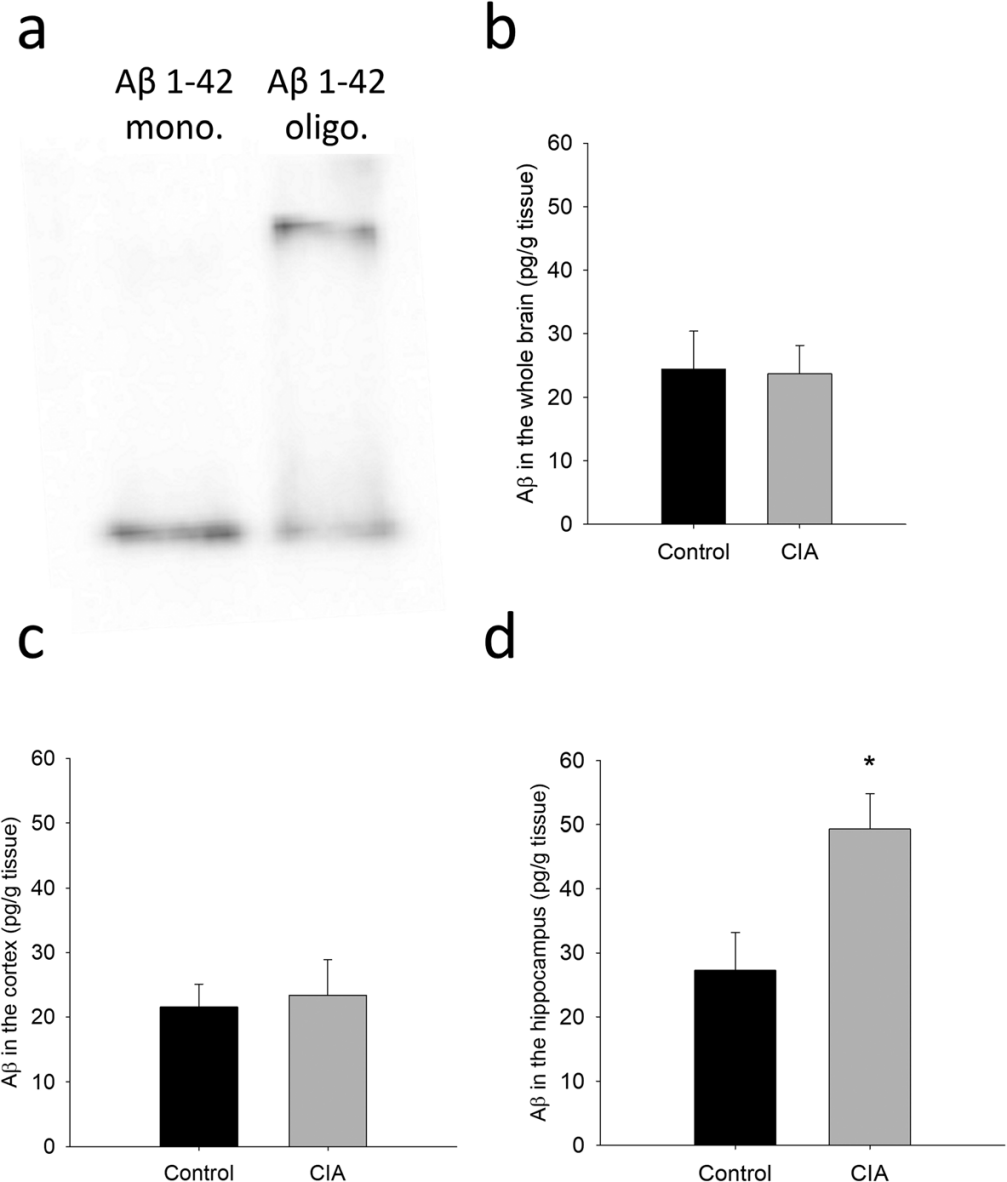
Increased blood-to-brain influx of monomeric Aβ42 in CIA rats. **a** Immunoblots of human Aβ42 monomer (left lane) and human Aβ42 oligomer (right lane). **b**-**d** The levels of Aβ42 in the whole brain (**b**), the cortex (**c**), and the hippocampus (**d**) following intravenous administration of human monomeric Aβ42 to CIA and control rats. The levels of Aβ42 was shown as Aβ42 (pg) divided by the weight of extracted tissues (g). The data are given as the mean ± SEM of 3-6 animals. *P < 0.05 compared with controls

To evaluate the impacts of P-gp and RAGE on BBB transfer of Aβ in CIA rats, human monomeric Aβ42 was injected intravenously and brain levels of Aβ were determined. Before the injection, Aβ42 was pre-disaggregated with HFIP to monomerize the peptide (Fig. 6a). Fifteen minutes after the intravenous administration, the levels of Aβ42 in the whole brain, the cortex, and the hippocampus were examined. The results showed that Aβ42 levels in the whole brain and the cortex were comparable between CIA rats and controls (Fig. 6b and c). However, in the hippocampus, the level of Aβ in CIA rats was about 1.8-fold higher than that in controls (*P* < 0.05) (Fig. 6d). This finding indicates an increased influx of Aβ from the blood to the hippocampus of CIA rats.

### Reduced plasma clearance of Aβ in CIA rats

**Fig. 7:**
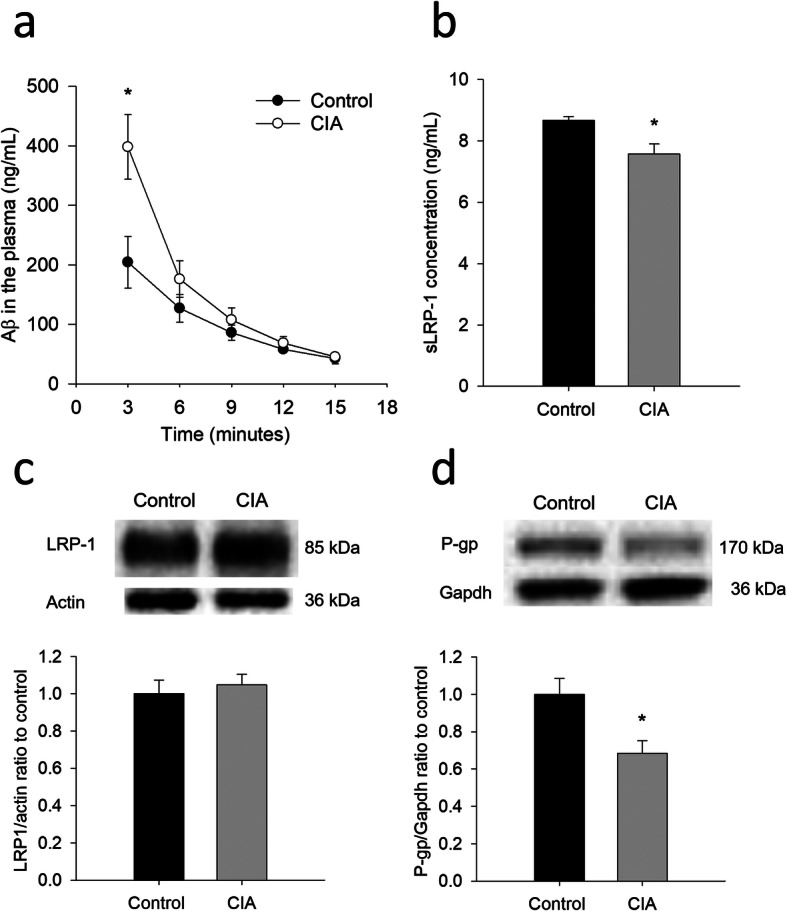
Reduced plasma clearance of Aβ42 in CIA rats. **a** Plasma concentrations of human Aβ42 following single intravenous injection in CIA rats and controls. **b** Plasma soluble LRP1 (sLRP1) levels in CIA rats and controls. **c** and **d** Immunoblotting and the quantitative densitometric analyses of hepatic LRP1 (**c**) and P-gp (**d**). The data are given as the mean ± SEM of 3-6 animals.*P < 0.05 compared with controls

As peripheral clearance of Aβ has been proposed to be implicated in the progression of AD [47], plasma concentrations of Aβ were measured. Following an intravenous injection of human Aβ42, plasma level of Aβ42 was significantly higher in CIA rats than in the controls at the first collection time (i.e., 3 min after the injection) (Fig. 7a). The systemic exposure of Aβ, represented by the total AUC, was also significantly higher in CIA rats (4092 ± 999 min × ng/mL) than in the controls (2397 ± 450 min × ng/mL) (*P* < 0.05). Further evaluation showed that the plasma clearance of Aβ was significantly lower in CIA rats (25 ± 6 mL/min) than in the controls (47 ± 8 mL/min) (*P* < 0.01).

Given that the factors contributing to peripheral clearance of Aβ may include sLRP-1, hepatic LRP-1, and hepatic P-gp [26], the expression of these proteins was also examined. As shown in Fig. 7b, plasma sLRP-1 levels were slightly but significantly decreased in CIA rats, compared with the controls. While the expression of hepatic LRP-1 was not significantly changed (Fig. 7c), the expression of hepatic P-gp was significantly decreased in the CIA rats (Fig. 7d).

## Discussion

In addition to synovial inflammation and cartilage damage, systemic inflammation is a hallmark of RA that can lead to altered gene expression in many tissues [21, 49] and contribute to neurodegeneration [30]. Hippocampal inflammation has been reported in patients with RA and in experimental RA [1]. Also, higher incidences of AD have been reported in patients with RA [5, 45]. Although the underlying links between RA and AD are to be clarified, many studies have indicated that BBB permeability may be altered in RA [36]. BBB dysfunction has been linked to the pathogenesis of many neurodegenerative diseases, including AD [42]. To date, the impacts of RA on BBB function, especially on BBB transport of Aβ, remain to be explored. BBB transport of Aβ may involve several pathways, including RAGE, LRP-1, and P-gp. Although an increased expression of RAGE has been identified in synovial tissue macrophages of patients with RA [40], it is not clear whether BBB expression of RAGE and others (e.g., LRP-1 and P-gp) is altered in RA. The present study showed that, in addition to neuroinflammation, BBB integrity was significantly decreased in CIA rats. The expression of both RAGE and P-gp, but not of LRP-1, was significantly altered in brain microvessels of CIA rats. Notably, much higher expression of RAGE was identified at brain arterioles of the hippocampus of CIA rats. Following intravenous administration, brain influx of Aβ was significantly higher in the hippocampus of CIA rats than in the controls; plasma clearance of Aβ was reduced in CIA rats. These findings indicate that RA plays a role in the disposition, including BBB transport and peripheral clearance, of Aβ.

Brain extracellular accumulation of Aβ is one of the hallmarks in AD, but the deposition of Aβ occurs much earlier than the onset of clinical symptoms [12]. Thus, an imbalance between production and clearance of Aβ can be an initiating factor for AD [39]. Given that only about 5% of AD patients are familial cases with an overproduction of Aβ, impaired Aβ clearance may play an important role in relation to AD pathogenesis in sporadic or late-onset AD, the most common form of AD [24, 46]. As Aβ is produced in both the brain and the periphery, the transport of Aβ across the BBB is bidirectional. Although the relative contribution of peripheral and central Aβ to the plaques is unclear, peripheral Aβ can enter the brain, forming the plaque and causing Aβ-related pathologies [4, 8]. In terms of Aβ transporting proteins, the changes in the expression and/or function of P-gp, RAGE, and LRP-1 were observed in AD patients [6, 44]. It was also demonstrated that the expression of RAGE was increased in brain microvessels of patients with cerebral amyloid angiopathy (CAA), a cerebrovascular dysfunction leading to cognitive impairment [33]. Thus, the finding that the expression of both RAGE and P-gp was increased in brain microvessels of CIA rats (Figs. 4 and 5) may provide a link between RA and abnormal amyloid deposition in the brain. However, RAGE and P-gp are responsible for brain influx and efflux of Aβ, respectively [18, 43, 52]. The impacts of increased RAGE and P-gp on Aβ transfer seem to be contradictory. Nonetheless, following an intravenous administration, our results showed that the level of the administrated Aβ was higher in the hippocampus, but not in the cortex, of CIA rats than in the controls, suggesting higher influx of peripheral Aβ to the hippocampus in CIA rats. This finding is consistent with the expression of RAGE in the brain, in which the increased RAGE expression was identified at the capillaries and, especially, at the arterioles located in the hippocampus (Figure 5). This is also consistent with the findings in patients with AD, in which higher level of RAGE was identified in the hippocampus than in the cortex [6]. Since the hippocampus is considered to be vulnerable in the early stage of AD [27], the increase in vascular RAGE expression and Aβ influx in the hippocampus in RA deserves further attention.

In addition to the roles of BBB on brain homeostasis of Aβ, sLRP-1 can sequester 70-90% Aβ in the blood, preventing it from entering the brain via the RAGE and enabling reductions in Aβ pathology [37]. It has been suggested that impairments in peripheral sLRP-mediated Aβ binding may serve as an early biomarker for mild cognitive impairment preceding AD-type dementia [38]. Also, the liver was suggested to be a major organ contributing to peripheral clearance of Aβ [9]. Hepatocytes can uptake Aβ from the blood through hepatic LRP-1 and excrete Aβ to biliary duct by P-gp [25]. Thus, the decreased expression of sLRP-1 (Fig. 7b) and hepatic P-gp (Fig. 7d) may cause an increase in plasma levels of Aβ in CIA rats. In line with this, CIA rats had higher plasma Aβ42 at initial phase, following an intravenous administration of Aβ (Fig. 7a). Similar findings have been reported by others: the effects of insulin on hepatic clearance of plasma Aβ only occurred at the initial phase following intravenous administration of Aβ [41]; plasma level of Aβ in AD transgenic APP/PS1 mice differed from that of controls at the initial phase [14]. This is probably because higher plasma levels of Aβ at the initial phase, but not the lower levels at the later phase, may saturate the elimination system (e.g., sLRP-1) involved in the plasma clearance of Aβ. Corroboratively, the plasma clearance of Aβ was significantly lower in CIA rats than in the controls. In terms of Aβ clearance in the brain, in addition to the BBB pathway, the roles of glymphatic and meningeal lymphatic pathways are worth an attention [22]. Further study is required to elaborate the impacts of RA on glymphatic–lymphatic drainage of Aβ.

While neuroinflammation and BBB dysfunction resulted from RA may suggest its susceptibility to the pathogenesis in AD, some studies have reported different results. For example, the induction of RA may provide protective effects on transgenic AD animal models: decreased Aβ accumulation was observed in CIA APP/PS1 transgenic mice [29]; lower tau pathology was observed in CIA tau-transgenic mice [19]. These studies seem to suggest that familial AD may be somehow beneficial from the inflammatory responses caused by RA, rather than the impacts of RA on the pathogenesis of sporadic AD.

## Conclusions

The induction of RA not only caused neuroinflammation but also altered the integrity and function of the BBB in rats. The changes in the expression of RAGE at brain blood vessels in the hippocampus can result in imbalanced BBB clearance of Aβ in RA. These findings suggest that RA may be implicated to the pathogenesis of amyloid deposition in the brain.

## Supplementary Information

**Additional file 1: Supplementary Table 1.** Primer sequences for RT-qPCR analysis. **Supplementary Figure 1.** Evaluation of collagen-induced arthritis (CIA) rats. **a** and **b** The change of right (**a**) and left (**b**) paw volume of control and CIA rats within 17 days following the first immunization (n=10). **c** The body weight change of control and CIA rats within 17 days following the first immunization (n=10). The data are the mean ± SEM. The asterisk indicates *P* value < 0.05 compared to the saline injected control group.

## Data Availability

The datasets used and/or analyzed during the current study are available from the corresponding author on reasonable request.

## Abbreviations

Aβ: Amyloid beta
AD: Alzheimer’s disease
BBB: Blood-brain barrier
CIA: Collagen-induced arthritis
Gfap: Glial fibrillary acidic protein
Iba-1: Ionized calcium-binding adapter molecule-1
IL-1β: Interleukin-1β
IL-6: Interleukin-6
LRP-1: Low-density lipoprotein receptor-related protein 1
MMP: Matrix metalloproteinase
P-gp: P-glycoprotein
RA: Rheumatoid arthritis
RAGE: Receptor of advanced glycation end product
sLRP-1: Soluble low-density lipoprotein receptor-related protein 1
TNF-α: Tumor necrosis factor-alpha
ZO-1: Zonula occludens-1
