# Supplementary material for: Changes of blood-brain-barrier function and transfer of amyloid beta in rats with collagen-induced arthritis
Source: J Neuroinflammation. 2021 Jan 30;18:35. doi: 10.1186/s12974-021-02086-2 (PMC7847579; doi:10.1186/s12974-021-02086-2)
Supplement: Supplementary file 1 — Additional file 1: Supplementary Table 1. Primer sequences for RT-qPCR analysis. Supplementary Figure 1. Evaluation of collagen-induced arthritis (CIA) rats. a and b The change of right (a) and left (b) paw volume of control and CIA rats within 17 days following the first immunization (n=10). c The body weight change of control and CIA rats within 17 days following the first immunization (n=10). The data are the mean ± SEM. The asterisk indicates P value < 0.05 compared to the saline injected control group. [file 12974_2021_2086_MOESM1_ESM.pdf]

Supplementary Table 1. Primer sequences for RT-qPCR analysis

Genes		Primer sequence	References
rat *IL-1β*	Forward	5-‘CACCTCTCAAGCAGAGCACAG-3’	1
	Reverse	5-‘GGGTTCCATGGTGAAGTCAAC-3’	
rat *IL-6*	Forward	5-‘TCCTACCCCAACTTCCAATGCTC-3’	1
	Reverse	5-‘TTGGATGGTCTTGGTCCTTAGCC-3’	
rat *TNF-α*	Forward	5-‘AAATGGGCTCCCTCTCATCAGTTC-3’	1
	Reverse	5-‘TCTGCTTGGTGGTTTGCTACGAC-3’	
rat *Gapdh*	Forward	5-‘CAACTCCCTCAAGATTGTCAGCAA -3’	2
	Reverse	5-‘GGCATGGACTGTGGTCATGA -3’	

References

1. Peinnequin A, Mouret C, Birot O, et al. Rat Pro-inflammatory cytokine and cytokine related mRNA quantification by real-time polymerase chain reaction using SYBR green. BMC Immunol. 2004;5:3.

2. Jewett M, Dickson E, Brolin K, Negrini M, Jimenez-Ferrer I, & Swanberg, M. Glutathione S-Transferase alpha 4 Prevents Dopamine neurodegeneration in a rat alpha-synuclein Model of Parkinson’s Disease. Front Neurol. 2018;9:222.

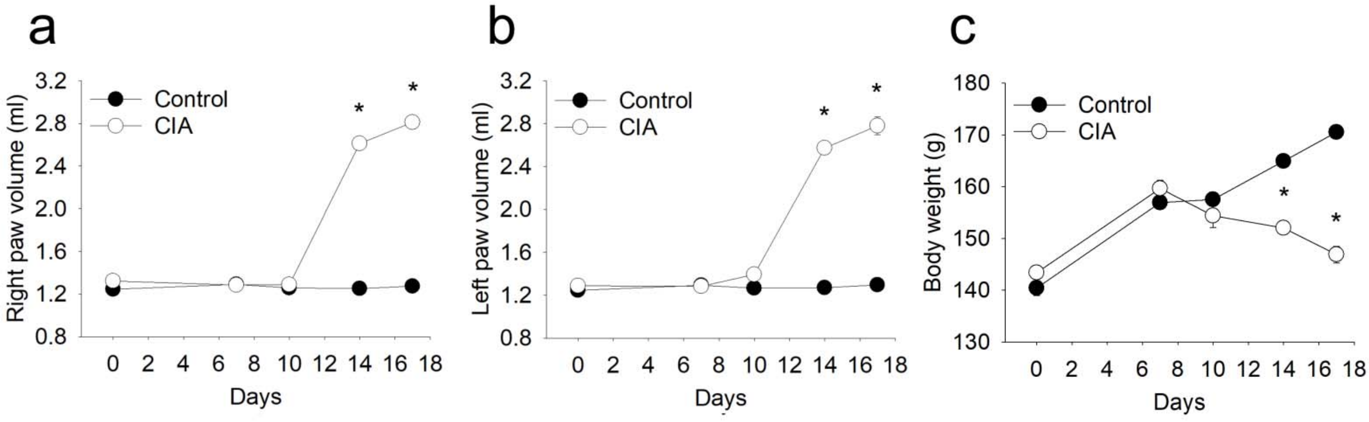

Supplementary Figure 1. Evaluation of collagen-induced arthritis (CIA) rats. **a** and **b** The change of right (**a**) and left (**b**) paw volume of control and CIA rats within 17 days following the first immunization (n=10). **c** The body weight change of control and CIA rats within 17 days following the first immunization (n=10). The data are the mean ± SEM. The asterisk indicates *P* value < 0.05 compared with the saline injected control group.
